# A semi–automatic system for subsampling heterogeneous foods

**DOI:** 10.1155/S1463924680000053

**Published:** 1980

**Authors:** J. F. Brown, H. T. Slover

**Affiliations:** Nutrient Composition Laboratory Nutrition Institute, Human Nutrition Center Science and Education Administration United States Department of Agriculture Beltsville Maryland 20705 USA


### A semi-automatic system for subsampling heterogeneous foods

J.F. Brown and H.T. Slover
*Nutrient Composition Laboratory, Nutrition Institute, Human Nutrition Center, Science and Education Administration, United States Department of Agriculture, Beltsville, Maryland 20705, USA.*

### Introduction

Chemical analysis of foods is preceded by subsampling the original sample. Reasonably homogeneous foods may present few problems, but the subsampling of heterogeneous materials requires care to assure meaningful analytical results. One effective solution to the problem, where sample size permits, is to homogenize the entire food item and subsample the homogenate. The success of this homogenization, the maintenance of homogeneity during subsampling and the protection of subsamples from degradation are crucial to the validity of subsequent nutrient determinations. For some time in the authors' laboratory, heterogeneous consumer food items, such as franchise hamburgers, french-fried potatoes and pastries, have been sampled after making homogeneous slurries in a Sorval* Model 17105 Omni-Mixer (Du Pont Company, Wilmington, DE 19898).

Briefly, the manual procedure for slurry preparation and subsampling has been to weigh the sample in an Omni-Chamber, to add a predetermined quantity of water containing antioxidants, to displace chamber air with nitrogen and to homogenize. Often it was necessary to add additional water to thin the slurry, cover again with nitrogen and continue homogenization. Following homogenization, the chamber and cover assembly were dismounted from the motor, the cover assembly was removed and subsamples were scooped out into small containers. Taking replicate subsamples manually is a slow and tedious process during which significant moisture loss and oxidation may occur. It is often further complicated by the need to rehomogenize samples which tend to separate. During each homogenization, sample is retained inside the shaft assembly; it is therefore necessary to completely disassemble it for proper cleaning and to prevent any cross contamination between samples.

Many homogenizers are commercially available, but none can be readily automated, or has the combined capabilities for large particle size reduction, simultaneous homogenization and subsampling, inert atmosphere sample protection and cleaning without disassembly. Consequently, a system has been developed in the authors' laboratory for preparing and subsampling slurries of food items based on a modified Omni-Mixer. The system is easily automated and operates faster providing more uniform results than manual methods. Although the system was devised to explore the feasibility of fully automatic sample preparation, it is valuable as a stand-alone unit where multiple representative aliquots of heterogeneous materials are needed. Details of the system's construction and operation are presented in this paper with some experimental results illustrating its performance. Its limitations and potential are also discussed.

### Principles of operation

Preliminary experiments in the authors' laboratory with complex foods showed that the sample size reduction and mixing, such as provided by the Omni-Mixer at viscosities near 3000 centipoise, yield analytically homogeneous slurries. Samples containing sensitive nutrients may be processed without damage when degassed solvents, antioxidants and inert atmospheres are used. For slurries that tend to separate, mixing must be continued during subsampling, and the flow of slurry in the sample lines must be maintained to provide representative subsamples. Disassembly of the unit for cleaning was unnecessary when the slurry was prevented from entering the drive shaft assembly and when all working surfaces could be cleaned by turbulent wash solution.

The six operating states of the slurry subsampling system are illustrated in Figure 1. The machine states are designated: Load/Unload, Dilute, Homogenize, Bypass, Dispense and Wash. Each state is activated by a single switch that is multiplexed to select relays and solenoid valves. The Omni-Mixer and solvent dispenser are manually controlled. Details of the modified Omni-Mixer are shown on Figure 2. Figure 3 is a diagram of the complete system.

In practice, a 450 ml Omni-Chamber is weighed, sample is added, and the combined weight is determined. The system is then switched to 'Load/Unload', which corresponds to Figure 1(a), and secured to the chamber. In this state, nitrogen is drawn from the dispenser head through the subsample by the application of a vacuum at the adaptor recess (Figure 2). The nitrogen clears any material from the slurry line and holds the sample in an inert atmosphere. For samples which require the addition of fluid to reach proper viscosity during homogenization, the system is switched to 'Dilute'. Known volumes of solvent are then pumped into the chamber through the shaft assembly, as shown in Figure 1 (b), by keying an accurately calibrated syringe pump. When a predetermined amount of solvent has been added to the chamber, the 'Homogenize' state is activated, and the mixer is turned to full power. This state is illustrated in Figure 1 (c). During homogenization, nitrogen flows through the shaft assembly, into the chamber and out through the adaptor recess and vacuum solvent port. In this way contamination of the shaft assembly is prevented and the sample is protected. If after several minutes the motor fails to reach a specified RPM, additional solvent can be added by returning the system to 'Dilute' and adding solvent from the syringe pump until the desired RPM is indicated.

After the homogenization is complete and the slurry is of the proper viscosity, the motor power is reduced to 25%. A flow of slurry is established between the homogenization chamber and dispenser head by nitrogen pressure and from the dispenser head to the bypass container, by a vacuum. When the slurry lines are wetted and a uniform flow obtained, subsamples may be taken by switching from 'Bypass' to 'Dispense' until the desired amount of subsample has been dispensed. This is shown in Figures 1d and 1e. Returning the system to 'Bypass' stops the subsampling process and diverts the flow of slurry back to the bypass container. Multiple subsamples may be taken by alternating between the 'Bypass ' and 'Dispense' modes. In the 'Wash' state, cleaning solution is drawn through the shaft assembly by a vacuum

** Mention of a trade-mark or proprietary product does not constitute a guarantee or warranty of the product by the U. S. Department of Agriculture, and does not imply its approval to the exclusion of other products that may also be suited.*

applied at the adaptor recess. This is shown in Figure 1f. This filling operation is carried out with the mixer at 25% power. When the chamber is full of wash solution, the system is switched to 'Bypass', the mixer turned to full power, and the syringe pump triggered. In this way, all interior surfaces are washed and waste is purged into the bypass container. After several 'Wash-Bypass' cycles, the system is switched to Load/ Unload', the empty Omni-Chamber is removed, and a new sample can be introduced.

### Instrumentation

The Omni-Mixer was modified to provide four access ports and to withstand moderate operating pressures. For details of the modified Omni-Mixer refer to Figure 2. The coupling adaptor was beveled so that O-rings could be fitted against both the motor casing and the chamber lid. A teflon flap seal was pressed between the motor and rubber coupling. The upper set screw in the rubber coupling was magnetized, and the coupling adaptor was drilled and tapped adjacent to the set screw to accept an inductance sensor.

Three holes were drilled through the chamber lid to match the slurry, lower recess and upper well ports in the teflon adaptor. The adaptor was sealed against the chamber and shaft assembly by O-rings and was configured to permit nitrogen, solvent or wash solution to enter a well in the top of the adaptor. A recess in the bottom of the adaptor allows a vacuum to be applied to the homogenization chamber and also the solvent to be pumped through the recess for cleaning purposes. A teflon tube was fitted on the drive shaft between the rulon bearings to reduce the dead volume in the shaft assembly. The flow of nitrogen, dilution solvent or wash solution through the shaft assembly was facilitated by drilling a hole above the lower rulon bearing and enlarging the upper hole which is at the bottom of the teflon adaptor well. The Omni-Mixer knives are, as supplied, blunt on the leading edges, and collagen fibers wound around the knives and shaft assembly when raw meats were homogenized. Sharpening the leading edges enabled raw meats to be processed without difficulty.

During the 'Bypass' and 'Dispense' modes of operation, slurry flow is controlled by the chamber valve and bypass valve. These were machined from teflon rod and moulded teflon tube and are pneumatically actuated. O-rings at both ends of the slider prevent control gas from entering the slurry lines; however, only press fitted teflon surfaces separate the input and output lines when the valves are closed. Both valves are identical except for a cross drilling in the bypass valve that permits the tube to the bypass chamber to be emptied while a subsample is being dispensed.

The dispenser head was designed in an open, three port venturi configuration and machined from teflon rod. Figure 4 illustrates its construction. As slurry flows through the dispenser head in the 'Bypass' mode, a partial vacuum in the bypass line and the slurry's own momentum prevent unintentional dispensing of sample. Any material retained in the subsample orifice is drawn into the bypassed slurry or wash flow. Nitrogen flows through a hole next to the subsample orifice to protect the dispensed subsample and to fill the homogenization chamber as it is being loaded with sample. The angle between dispenser head ports was chosen to minimize turbulence in the bypassed flow yet optimize venturi suction at the subsample orifice.

Proper operation of the system depends on the final slurry viscosity, which is measured as a function of mixer RPM. Its speed is measured using a Teledyne-Philbrick 4722 integrated circuit frequency to voltage converter pulsed by an induction

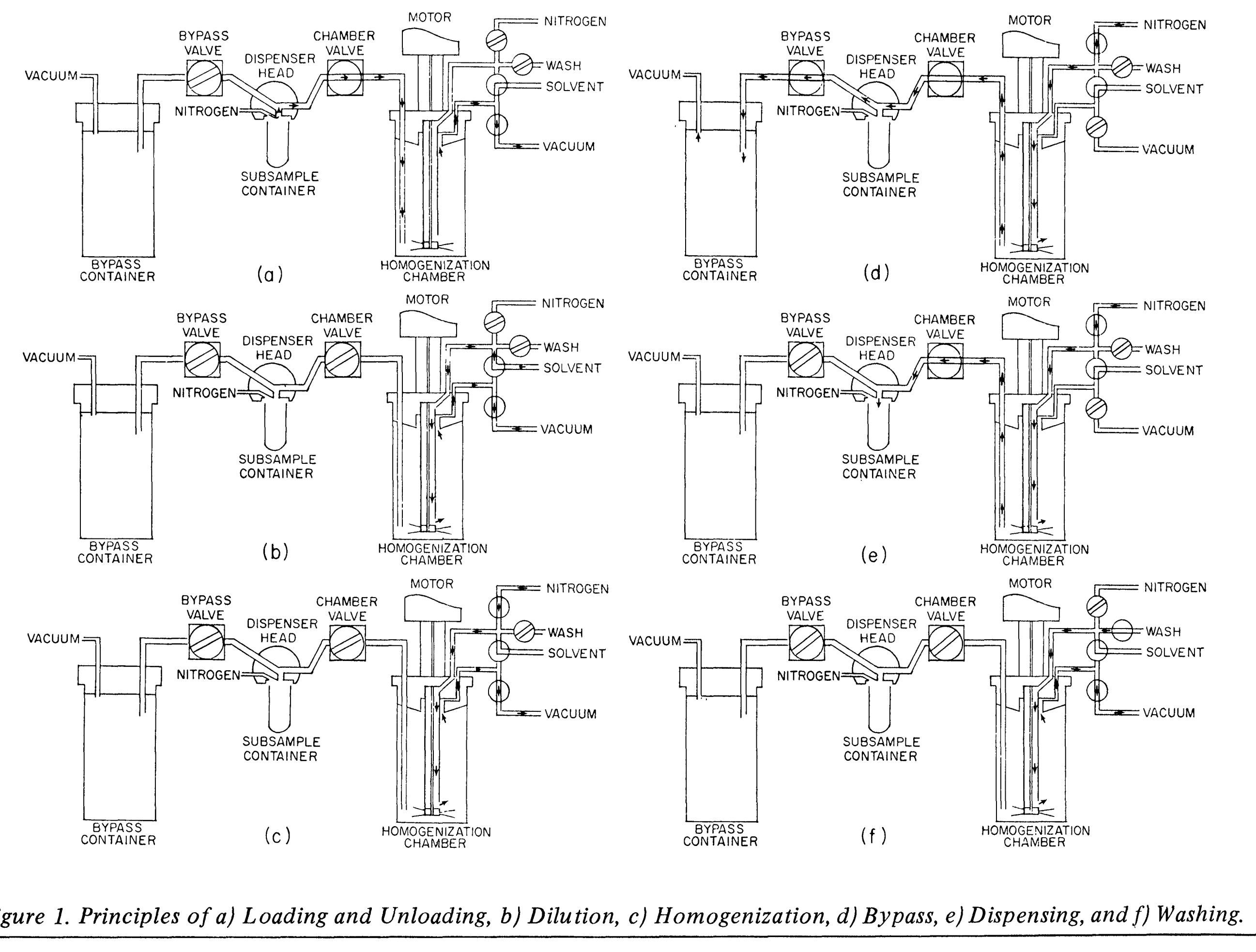

*Figure 1. Principles of a) Loading and Unloading, b) Dilution, c) Homogenization, d) Bypass, e) Dispensing, and f) Washing.*

*Table 1. Coefficients of variation for moisture and total lipid data from various complex foods.*

| | Coefficients of variation | | | | | |
|---|---|---|---|---|---|---|
| | Moisture | | | Total lipid | | |
| Sample | N | Manual | Automatic | N | Manual | Automatic |
| Hamburger & bun | 4 | 0.22 | 0.10 | 11 | 3.15 | 2.53 |
| Raw hamburger | – | – | – | 4 | 0.85 | 0.90 |
| French fries | 4 | 1.18 | 1.05 | 8 | 3.38 | 2.80 |
| Italian dressing | 4 | 1.00 | 0.23 | 4 | 0.87 | 0.51 |
| Pastry | 4 | 2.43 | 0.47 | 7 | 1.10 | 0.59 |

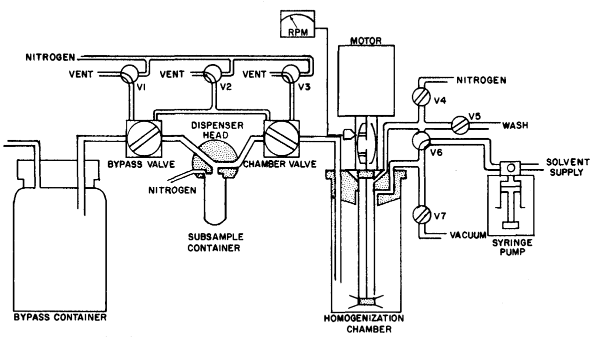

*Figure 3. Schematic of Subsampling system.*

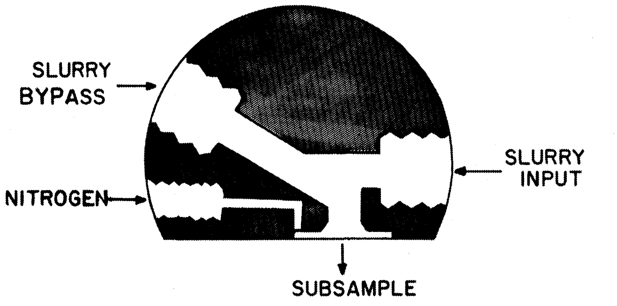

*Figure 4. Cross-section of dispenser head.*

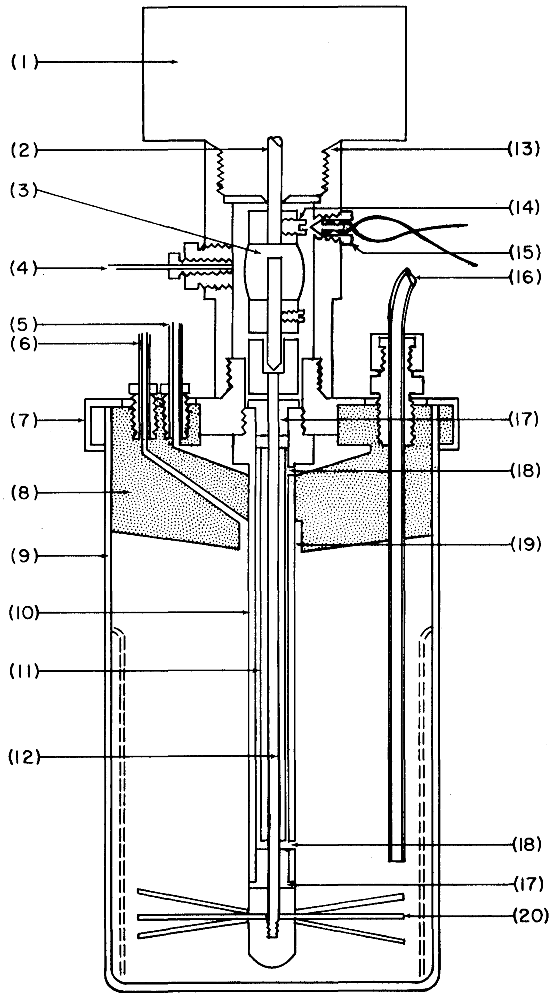

*Figure 2. Diagram of modified Omni-Mixer assembly. 1) motor, 2) motor shaft, 3) rubber coupling, 4) nitrogen, 5) nitrogen/wash/solvent, 6) vacuum/solvent, 7) cover, 8) teflon adapter, 9) chamber, 10) shaft assembly, 11) teflon tube, 12) drive shaft, 13) coupling adapter, 14) magnetic screw, 15) magnetic transducer, 16) sample tube, 17) rulon bearings, (2), 18) 1 mm hole (2), 19) adapter recess, 20) blades.*

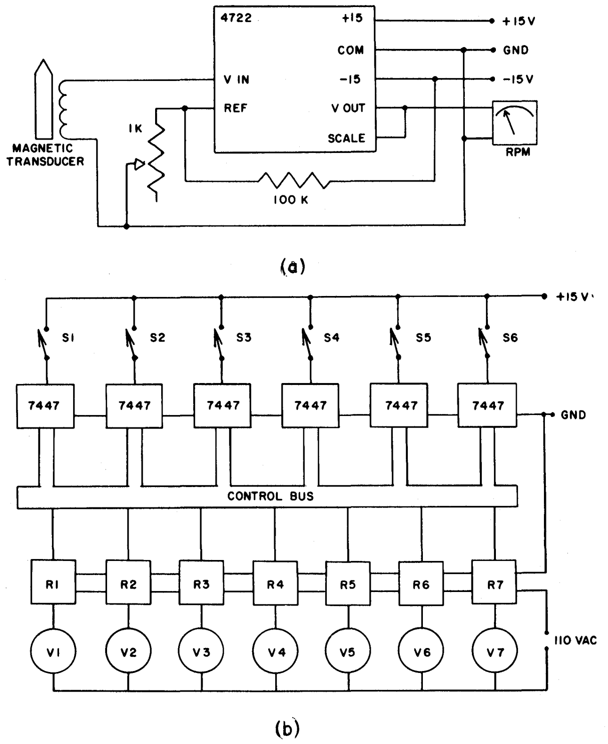

*Figure 5. Schematic of control circuitry, a) tachometer, b) multiplexer, relays and valves. (Teledyne Philbrick, Dedham, MS 0Z026; all resistors ¼ watt, 10%; National Semiconductor Corporation, Santa Clara, CA 95051; Angar Scientific Corporation; East Hanover, NJ 07936.*

sensor in the coupling adaptor adjacent to the magnetic set screw. Figure 5a is a schematic of the tachometer. The potentiometer can be adjusted to give full meter deflection for any RPM. Nitrogen and solutions are controlled by Angar scientific three-way model 250 TFE solenoid valves and the vacuum is controlled by an Asco two-way model 104R solenoid valve. All valves are multiplexed from six switches to facilitate changing machine states, as shown in Figure 5b. Each switch fully enables a single National 7447 decoder/driver. Jumper wires connect to select Teledyne P/N 601-1401P relays, which activate the Angar valves. The definition of any state can be altered by simply changing jumper wires between the relays and the individual decoder/drivers.

Detailed schematics and operating instructions are available upon request.

### System evaluation

Slurries of raw and franchise hamburger, salad dressing, french fries and pastries were analyzed from samples prepared and subsampled by both manual methods and the dispenser system. Samples requiring dilution to obtain proper viscosity were diluted with a solution of 4g pyrogallol per litre of distilled water. The apparatus was cleaned with a 20% solution (v/v) of ethanol and distilled water.

The dispenser system was evaluated and compared with manual subsampling on the basis of precision in determination of moisture (1) on replicate 2g subsamples, and of total lipid (2) and fatty acids (3) on chloroform-menthol extracts of replicate 10g subsamples. The result of these comparisons are shown in Table 1. The data show that precision was improved by use of the dispenser system. No significant differences were found in moisture, total lipid or the absolute fatty acid compositions between subsamples automatically dispensed and taken manually. The thoroughness of the cleaning cycle was determined by rinsing the disassembled unit with a mixture of chloroform and methanol, partitioning with water and measuring the total lipid in the resulting lower phase. There was no significant cross contamination between samples after four 'Wash-Bypass' cleaning cycles (Figure 6).

With the present system the feasibility of automatic slurry handling has been demonstrated; this improves subsample uniformity and reduces the operation time for staff. With the system described here, sample charges ranging from a maximum of 400 ml to a minimum of 50 ml can be processed. At about 20 psig chamber pressure, subsamples may be taken at the rate of 1 ml per second in aliquots as small as 0.2 ml. Most samples can be homogenized in 5 minutes, and the system can be washed in 3 minutes.

The system is limited primarily because a gas pressure must be used to pump the homogenate. Adjustments in nitrogen pressure are sometimes necessary to offset differences in the flow shear rate between slurries of similar viscosity, and time was not a reliable measure of subsample size. Where many small subsamples are required, typically 30% of the slurry is lost to the bypass chamber. In practice samples are collected and stored in inexpensive glass containers and transferred to stainless steel Omni-Chambers for homogenization and subsampling. It would be more efficient to store and homogenize samples in the same vessel, but glass would probably be incompatible with even the moderate operating pressures described. In order to permit the use of glass jars for sample collection, storage and homogenization, a peristaltic pump will be used to replace the gas pressure for fluid transport. Further plans for this device include the addition of microprocessor control for the operation of valves and pumps.

ACKNOWLEDGEMENT
We appreciate the assistance of C. S. Davis and E. Lanza in generating the data, and of P. P. Padovano in producing the drawings.

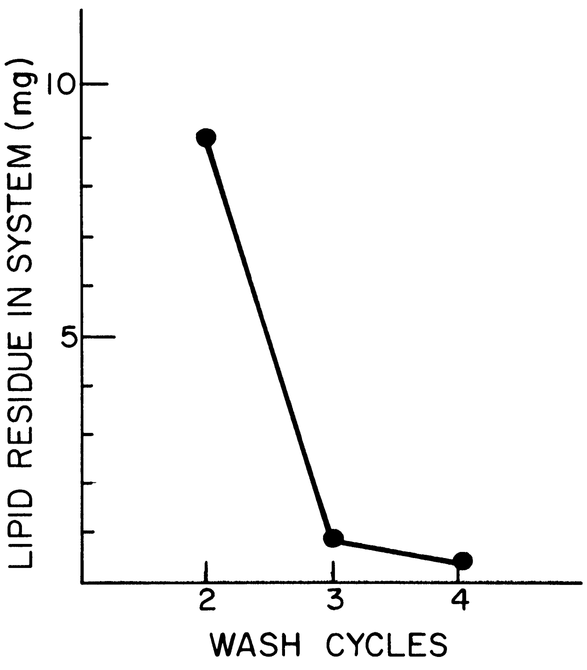

*Figure 6. Change over data showing milligrams of lipid residue in subsampling system after successive wash-purge cycles.*

REFERENCES
[1] *Official Methods of Analysis of the Association of Official Analytical Chemists,* William Horwitz, Editor 31.005 Eleventh Edition, 1970.
[2] Folch, J. and Stanley, G.H.S., *J. of Biol. Chem.,* (1957) **226:** 497-509.
[3] Slover, H.T. and Lanza, E., *J. Amer. Oil Chemist's Soc.,* (1978) 55: 265A. (Abstract).

